# Pyrethroid resistance status and co-occurrence of V1016G, F1534C and S989P mutations in the *Aedes aegypti* population from two dengue outbreak counties along the China-Myanmar border

**DOI:** 10.1186/s13071-024-06124-9

**Published:** 2024-02-27

**Authors:** Li Chen, Kemei Zhou, Jun Shi, Yuting Zheng, Xiaotao Zhao, Qingyun Du, Yingkun Lin, Xaioxiong Yin, Jinyong Jiang, Xinyu Feng

**Affiliations:** 1 Faculty of Life Science and Technology, Kunming University of Science and Technology, Kunming, China; 2 Yunnan International Joint Laboratory of Tropical Infectious Diseases, Yunnan Provincial Key Laboratory of Vector-Borne Diseases Control and Research, Yunnan Key Technology Innovation Team for Insect Borne Infectious Disease Prevention and Control, Yunnan Institute of Parasitic Diseases, Pu’er, China; 3 Lincang Center for Disease Control and Prevention, Lincang, China; 4 Gengma Center for Disease Control and Prevention, Gengma, China; 5 Dehong Prefecture Center for Disease Control and Prevention, Mangshi, China; 6 Ruili Center for Disease Control and Prevention, Ruili, China; 7 School of Global Health, Chinese Center for Tropical Diseases Research, Shanghai Jiao Tong University School of Medicine, Shanghai, 20025 China; 8 One Health Center, Shanghai Jiao Tong University, The University of Edinburgh, Shanghai, 20025 China

**Keywords:** *Aedes Aegypti*, Insecticide resistance, Pyrethroid, Knockdown resistance, Mutation

## Abstract

**Background:**

Over the past two decades, dengue fever (DF) has emerged as a significant arboviral disease in Yunnan province, China, particularly in the China-Myanmar border area. *Aedes aegypti*, an invasive mosquito species, plays a crucial role in transmitting the dengue virus to the local population. Insecticide-based vector control has been the primary tool employed to combat DF, but the current susceptibility status of *Ae. aegypti* to commonly used insecticides is unknown. Assessment of *Ae. aegypti* resistance to pyrethroid insecticides and an understanding of the underlying mechanisms of this resistance in the China-Myanmar border region is of significant strategic importance for effectively controlling the DF epidemic in the area.

**Methods:**

*Aedes aegypti* larvae collected from Ruili and Gengma counties in Yunnan Province were reared to adults in the laboratory and tested for susceptibility to three pyrethroid insecticides (3.20% permethrin, 0.08% lambda-cyhalothrin and 0.20% deltamethrin) by the standard WHO susceptibility bioassay. Genotyping of mutations in the knockdown gene (*kdr*), namely S989P, V1016G and F1534C, that are responsible for resistance to pyrethroid insecticides was performed using allele-specific PCR methods. A possible association between the observed resistant phenotype and mutations in the voltage-gated sodium channel gene (*VGSC*) was also studied.

**Results:**

*Aedes aegypti* mosquitoes collected from the two counties and reared in the laboratory were resistant to all of the pyrethroids tested, with the exception of *Ae. aegypti* from Gengma County, which showed sensitivity to 0.20% deltamethrin. The mortality rate of *Ae. aegypti* from Ruili county exposed to 3.20% permethrin did not differ significantly from that of *Ae. aegypti* from Gengma County (*χ*^2^ = 0.311, *P* = 0.577). By contrast, the mortality rate of *Ae. aegypti* from Ruili County exposed to 0.08% lambda-cyhalothrin and 0.20% deltamethrin, respectively, was significantly different from that of *Ae. aegypti* from Gengma. There was no significant difference in the observed KDT_50_ of *Ae. aegypti* from the two counties to various insecticides. Four mutation types and 12 genotypes were detected at three *kdr* mutation sites. Based on results from all tested *Ae. aegypti*, the V1016G mutation was the most prevalent *kdr* mutation (100% prevalence), followed by the S989P mutation (81.6%) and the F1534C mutation (78.9%). The constituent ratio of *VGSC* gene mutation types was significantly different in *Ae. aegypti* mosquitoes from Ruili and those Gengma. The triple mutant S989P + V1016G + F1534C was observed in 274 *Ae. aegypti* mosquitoes (60.8%), with the most common genotype being SP + GG + FC (31.4%). The prevalence of the F1534C mutation was significantly higher in resistant *Ae. aegypti* from Ruili (odds ratio [OR] 7.43; 95% confidence interval [CI] 1.71–32.29; *P* = 0.01) and Gengma (OR 9.29; 95% CI 3.38–25.50; *P* = 0.00) counties than in susceptible *Ae. aegypti* when exposed to 3.20% permethrin and 0.08% lambda-cyhalothrin, respectively. No significant association was observed in the triple mutation genotypes with the *Ae. aegypti* population exposed to 3.20% permethrin and 0.20% deltamethrin resistance (*P* > 0.05), except for *Ae. aegypti* from Gengma County when exposed to 0.08% lambda-cyhalothrin (OR 2.86; 95% CI 1.20–6.81; *P* = 0.02).

**Conclusions:**

*Aedes aegypti* from Ruili and Gengma counties have developed resistance to various pyrethroid insecticides. The occurrence of multiple mutant sites in *VGSC* strongly correlated with the high levels of resistance to pyrethroids in the *Ae. aegypti* populations, highlighting the need for alternative strategies to manage the spread of resistance. A region-specific control strategy for dengue vectors needs to be implemented in the future based on the status of insecticide resistance and *kdr* mutations.

**Graphical Abstract:**

**Figure:**
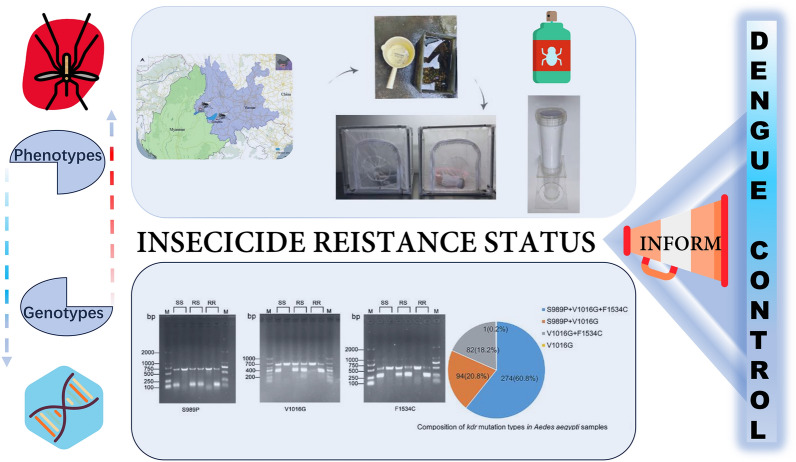

**Supplementary Information:**

The online version contains supplementary material available at 10.1186/s13071-024-06124-9.

## Background

Dengue fever (DF) is a mosquito-borne disease transmitted by *Aedes aegypti* or *Aedes albopictus* mosquitoes that carry the dengue virus. It is prevalent throughout tropical and subtropical countries and regions, with particularly severe outbreaks recorded in Southeast Asia and the Western Pacific regions [1]. China's Yunnan Province shares borders with Vietnam, Laos and Myanmar, with a border length of 2185.7 km with Myanmar [2]. DF is a major public health concern in Myanmar, where a total of 58,461 cases were reported between 2017 and 2019 [3, 4]. This high incidence rate of DF in Myanmar has resulted in multiple local outbreaks in other regions, including in Ruili and Gengma counties, Yunnan Province [5]. Approximately 5500 cases of DF have been reported in these counties, indicating that DF is a significant vector-borne disease with a substantial burden on public health [6–9]. The dominant mosquito species inhabiting Ruili and Gengma counties is *Ae. aegypti* [8, 10–12]. As a preventative measure aimed at combating the DF epidemic, pyrethroid insecticides have been applied consistently in an effort to effectively reduce the population density of *Aedes* mosquitoes and prevent the spread of the disease. However, this extensive and prolonged use of these insecticides has resulted in the emergence of resistance among local mosquito populations, posing a significant obstacle to the control of DF in the region.

Insecticide resistance in mosquitoes can be attributed to several mechanisms, including knockdown resistance (*kdr*), metabolic resistance, epidermal resistance and behavioral resistance [13]. Resistance to pyrethroid insecticides in *Ae. aegypti* is primarily attributed to mutations in kdr genes (*kdr*) and to metabolic detoxification mechanisms [14]. *Kdr* mutations result in alterations in the voltage-gated sodium channel proteins (*VGSC*), the target site of pyrethroids and organochlorines. These mutations reduce the binding affinity of pyrethroids to the *VGSC*, thereby reducing their effectiveness [15]. The most commonly observed mutation associated with pyrethroid resistance, V1016G, is found in the IIS6 region of the *Ae. aegypti VGSC* gene [16], with mutations also found at other sites in IIS6 region, such as G923V, L982W, S989P, V1016I, A1007G and I1011V/M. Another mutation, F1534C, in the IIIS6 region of the* VGSC* gene, has also been identified and is associated with pyrethroid resistance [16–18].

The phenomenon of *Ae. aegypti* mosquitoes developing resistance to pyrethroid insecticides has been noted across multiple countries and regions worldwide. Progress in researching this topic has been made in both China and the Greater Mekong Subregion (GMS), with pyrethroid resistance gaining significant attention from researchers in China in recent years. Several studies have investigated the prevalence and mechanisms of resistance in different regions of China. One study conducted in southern China found high levels of pyrethroid resistance in *Ae. aegypti* populations and identified the S989P, V1016G and F1534C mutations [19]. Another study conducted in Yunnan Province, southwest China, also reported high levels of pyrethroid resistance in *Ae. aegypti* populations, and identified the V1016G mutation as the primary resistance mechanism in this region. In addition to identifying single-point mutations, researchers have also detected new co-occurrence of mutations related to pyrethroid resistance in *Ae. Aegypti* [20, 21]. Several studies have shown that the presence of both V1016G and F1534C mutations in the same individual or population in Southeast Asian countries results in a higher level of resistance to pyrethroids [22–24].

Additionally, a study found evidence of co-occurrence of triple mutations (S989P, V1016G and F1534C), which resulted in even higher resistance to pyrethroids, as observed in *Ae. aegypti* populations from Myanmar [25]. These mutations in the *VGSC* gene of *Ae. aegypti* are believed to play a significant role in the development of pyrethroid resistance. However, it is important to note that resistance mechanisms can be complex and multifactorial, involving additional genetic variations and metabolic detoxification pathways.

In light of the development of insecticide resistance, data are asymmetric both within China and in the GSM countries. In-depth studies on insecticide resistance in *Ae. aegypti* are critical to controlling the spread of diseases transmitted by this mosquito species. Therefore, we have investigated the resistance status of two *Ae. aegypti* populations from Ruili and Gengma counties to three pyrethroid insecticides, namely permethrin, lambda-cyhalothrin, and deltamethrin. Molecular surveillance based on allele-specific PCR (AS-PCR) was utilized to confirm the presence of the commonly reported V1016G, F1534C and S989P mutations and to determine the presence of particular genetic variations or mutation combinations that contribute to pyrethroid resistance. Finally, the association between mutant and phenotype was statistically analyzed to explore the mechanisms of the resistance. The findings of the study will provide scientific evidence for the control of *Ae. aegypti* in Yunnan Province.

## Methods

### Mosquito collection and rearing

**Fig.1:**
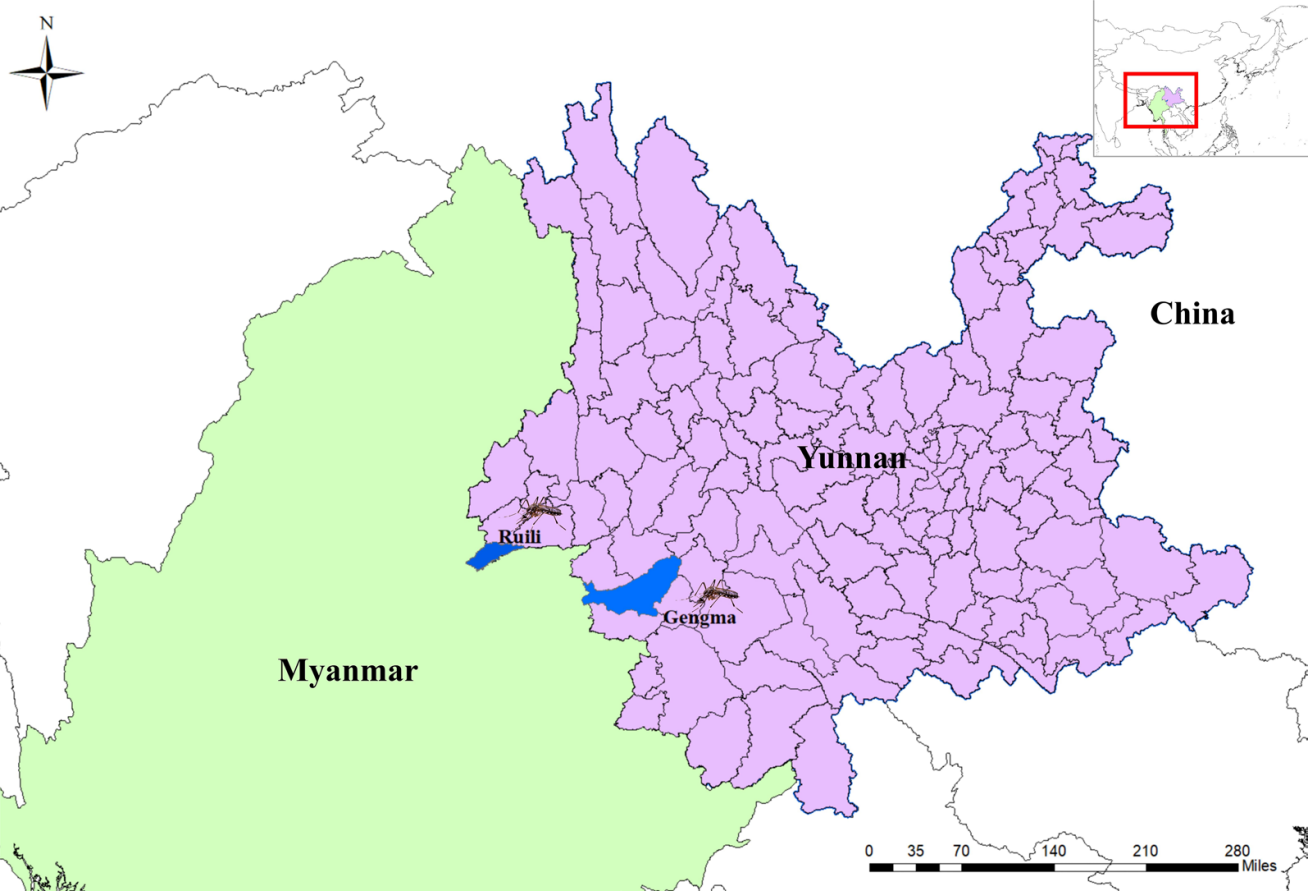
*Aedes aegypti* mosquito collection sites from two different urban areas of Yunnan Province, China. Map was created with the software ArcMap 10.1 in ArcGIS 10.1 (ESRI, Redlands, CA, USA)

The study was conducted in two different urban areas of Yunnan province, namely Ruili County of Dehong prefecture (24.0108°N, 97.8556°E) and Gengma County of Lincang City (23.5540°N, 99.0819°E). From September to December 2022, larvae and pupae of *Ae. aegypti* were collected from various artificial outdoor breeding sites, such as abandoned car tires, discarded containers and cans (Fig. 1). The collected larvae were collected in plastic containers, brought back to the laboratory and reared until they emerged into adults (F_0_). The rearing conditions consisted of a constant temperature of 26 ± 1 °C, relative humidity of 65 ± 5% and a dark:light cycles of 12:12 h. After emergence, the adult mosquitoes were identified to species level based on morphological characters.

#### WHO susceptibility test

The adult *Ae. aegypti* mosquito bioassay was conducted following the WHO susceptibility test protocol [26]. A total of 20–30 non-blood-fed female *Ae. aegypti* mosquitoes aged 3–5 days were used in the bioassays. These adults were exposed to impregnated paper with 3.20% permethrin, 0.08% lambda-cyhalothrin and 0.20% deltamethrin, respectively (all insecticides were provided by the Institute of Infectious Disease Prevention and Control, Chinese Center for Disease Control and Prevention), for 1 h. The diagnostic doses reported in this study refer to the values established by the China Centers for Disease Control and Prevention [27, 28]. Following exposure, the mosquitoes were transferred to separate holding tubes, and the number of deceased mosquitoes was tallied after a 24-h period. Probit regression analysis was used to determine the time (in minutes) taken for 50% of the test mosquitoes to be knocked down (KDT_50_) and 95% confidence interval (95% CI) of *Ae. aegypti* in Ruili County and Gengma County in response to different insecticides. After 24 h of recovery, the number of dead and live mosquitoes were recorded, and the mortality rate associated with each tested insecticide was calculated. Mosquitoes were considered to be deceased if they were unable to fly or had turned upside down regardless of slight leg tremors. Each insecticide was tested in three replicates, and control groups were set up. If the mortality rate in the control group was > 20%, the experiment was considered to be unsuccessful and to necessitate a restart. If the mortality rate in the control group was < 20%, the results had to be corrected using Abbott’s formula. S indicates susceptible, based on a mortality rate of ≥ 98%; M indicates suspected resistant, based on a mortality rate  90% to 98%; R indicates resistant, based on a mortality rate < 90% [29].

### Allele-specific PCR genotyping of *kdr* mutation

Following the bioassay, mosquito DNA was extracted according to the manufacturer's instructions (TianGen Biotech, Beijing; Art. No. 22011210T332). Two fragments of the* VGSC* gene (DII-S6 and DIII-S6) were amplified using allele-specific PCR (AS-PCR) [13, 19] sets (Additional file 1: Table S1) as previously described. The thermal cycling conditions used for the experiment consisted of an initial denaturation at 94 °C for 3 min; followed by 35 cycles of denaturation at 94 °C for 30 s, annealing at 51 °C or 60 °C for 30 s (51 °C for S989P, 60 °C for V1016G and F1534C) and extension at 72 °C for 60 s; with a final extension at 72 °C for 5 min. A negative control PCR tube was run, which contained all of the components of the AS-PCR except for DNA. After we conducted a thorough analysis and made precise modifications to the various factors involved in the experiment, the AS-PCR protocol displayed exceptional efficiency in precisely distinguishing between homozygous and heterozygous mosquitoes with regards to three crucial mutations, namely S989P, V1016G, and F1534C, with the application of 1.2% agarose gel electrophoresis.

### Data analysis

Microsoft Excel version 365 (Microsoft Corp., Redmond, WA, USA) was used to organize the data and calculate the mortality rates and *kdr* mutation rates of *Ae. aegypti* in response to the three types of pyrethroids. SPSS version 25.0 software (SPSS IBM, Armonk, NY, USA) was used for the statistical analysis that compared the mortality rates and *kdr* gene mutation rates at different sites, as well as the differences in resistance phenotype and sensitive phenotype *kdr* gene mutation rates of *Ae. aegypti* in Ruili and Gengma counties in response to different insecticides. Pearson’s Chi-square test (*χ*^2^) was applied to analyze differences in mortality rates and *kdr* gene mutation rates at different sites. Additionally, the *kdr* gene mutation rates for both resistant and susceptible phenotypes of *Ae. aegypti* to the three insecticides were assessed in Ruili and Gengma counties. *P* values < 0.05 were considered to indicate statistical significant difference. Odds ratios (ORs) and 95% CIs were estimated using Fisher’s exact test to assess the associations between *kdr* mutations and resistant phenotypes; at an OR > 1.0 and *P* < 0.05, the *kdr* mutation type was considered to be significantly associated with insecticide resistance.

## Results

### Species identification and sampling size

From September to December 2022, > 1000 larvae were collected from Ruili and Gengma; following emergence in the laboratory, 485 female mosquitoes were morphologically identified as *Ae. aegypti* and subsequently tested for insecticide susceptibility to 3.20% permethrin, 0.08% lambda-cyhalothrin and 0.20% deltamethrin, respectively.

### *Aedes aegypti* insecticide bioassays

The mortality rates of *Ae. aegypti* from Ruili County upon exposure to 3.20% permethrin, 0.08% lambda-cyhalothrin and 0.20% deltamethrin were 18.1%, 11.43% and 24.0%, respectively. The mortality rates of *Ae. aegypti* from Gengma County upon exposure to these three insecticides at the same concentrations were 21.9%, 36.1% and 100.0%, respectively. Based on the mortality rates, *Ae. aegypti* mosquitoes from the two counties were considered to be resistant to all of the tested pyrethroids, with the exception of *Ae. aegypti* from Gengma County, which showed sensitivity to 0.20% deltamethrin (Table 1). The mortality rate of *Ae. aegypti* from Ruili County exposed to 3.20% permethrin was not significantly different from that of *Ae. aegypti* from Gengma County (*χ*^2^ = 0.311, *df* = 1, *P* = 0.577). In contrast, the mortality rate of *Ae. aegypti* from Ruili County exposed to 0.08% lambda-cyhalothrin was 11.43%, which was significantly different from that of *Ae. aegypti* from Gengma County (*χ*^2^ = 12.93, *df* = 1, *P* < 0.001). The mortality rate of *Ae. aegypti* from Ruili County exposed to 0.20% deltamethrin was also significantly different from that of *Ae. aegypti* from Gengma County (*χ*^2^ = 118.40, *df* = 1, *P* < 0.001).

**Table 1:** The resistance status of *Aedes aegypti* from Ruili and Gengma counties to the three tested insecticides

Pesticides (concentration)	Sampling point	Number of mosquitoes tested	Number of knockdowns in 1 h (*N* mosquitoes)	Knockdown rate (%)	Statistical analysis	KDT_50_, in minutes (95% CI)	Number of deaths (*N* mosquitoes)	24-Hour mortality rate (%)	Resistance status^a^	Statistical analysis
Permethrin (3.20%)	Ruili	72	23	31.9	*χ*^2^ = 0.079, *df* = 1, *P* = 3.086	70.277 (63.133–86.847)	13	18.06	R	*χ*^2^ = 0.311, *df* = 1, *P* = 0.577
	Gengma	64	12	18.8		409.184 (151.079–19555.008)	14	21.88	R	
Lambda-cyhalothrin (0.08%)	Ruili	70	29	41.4	*χ*^2^ = 2.385, *df* = 1, *P* = 0.122	64.950 (59.577–75.552)	8	11.43	R	*χ*^2^ = 12.926, *df* = 1, *P* = 0.000*
	Gengma	97	29	29.9		100.955 (79.627–149.577)	35	36.08	R	
Deltamethrin (0.20%)	Ruili	75	51	68.0	*χ*^2^ = 1.312, *df* = 1, *P* = 0.252	47.100 (43.892–51.117)	18	24.0	R	*χ*^2^ = 118.402, *df* = 1, *P* = 0.000*
	Gengma	107	81	75.7		37.841 (34.874.-41.311)	107	100.0	S	
Control	Ruili	25	0	0	–^b^	/^c^	0	0	/	–
	Gengma	25	0	0		/	0	0	/	

*CI* Confidence interval* KDT*_*50*_ Time (in minutes) taken for 50% of the test mosquitoes to be knocked down,

*Statistical significant difference at *P* < 0.05

^a^S indicates susceptible, based on a mortality rate of ≥ 98%; R indicates resistant, based on a mortality rate  < 90%

^b^–, Not determined

^c^/, No results

**Fig. 2:**
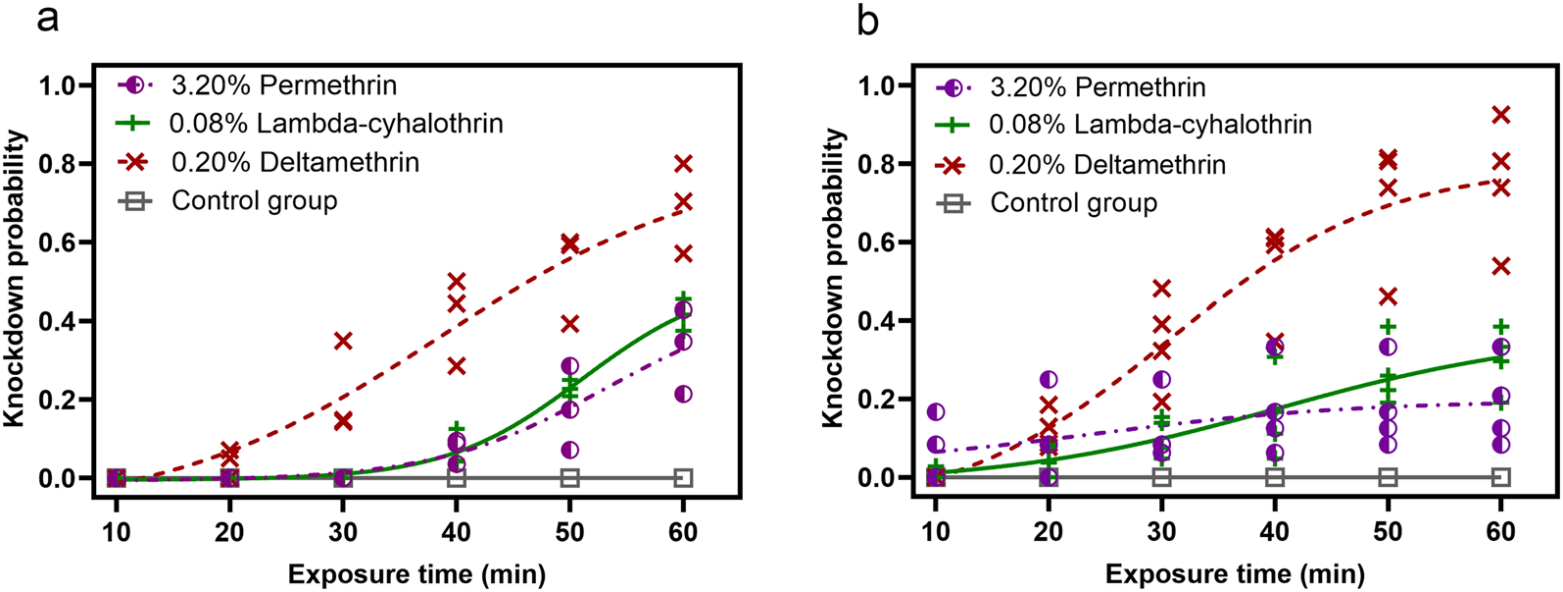
Knockdown times of *Aedes aegypti* from Ruili County (**a**) and Gengma County (**b**) following exposure to 3.20% permethrin, 0.08% lambda-cyhalothrin and 0.20% deltamethrin, respectively

The KDT_50_ and 95% CI of the two *Ae. aegypti* populations in Ruili and Gengma counties in response to different insecticides are shown in Fig. 2 and Table 1. There was no significant difference in the knockdown rates of *Ae. aegypti* from Ruili and those from Gengma showed following exposure to 3.20% permethrin, 0.08% lambda-cyhalothrin and 0.20% deltamethrin, respectively (Table 1). Exposure to 0.20% deltamethrin had a rapid knockdown effect on *Ae. aegypti* from both Gengma County and Ruili County. In contrast, the KDT_50_ of *Ae. aegypti* from Gengma County was much higher than the KDT_50_ of *Ae. aegypti* from Ruili County following exposure to permethrin (3.20%) and lambda-cyhalothrin (0.08%), respectively (Table 1).

### Identification of *kdr* mutations and genotypes in *Ae. aegypti*

**Fig. 3:**
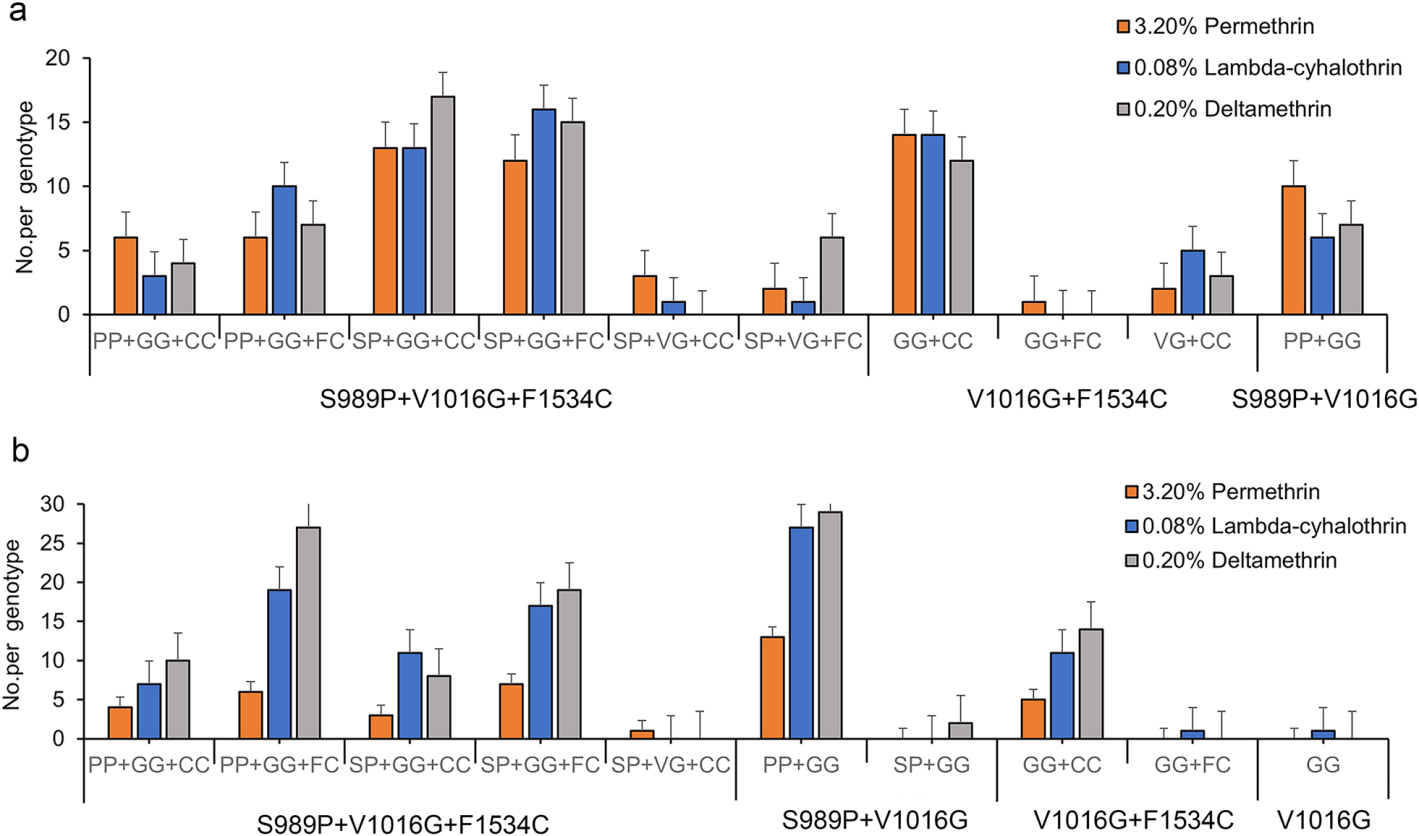
Different *kdr* mutations and genotypes in *Ae. aegypti* from Ruili County (**a**) and Gengma County (**b**), Yunnan Province.* kdr* Knockdown gene

Genomic DNA was obtained from 451 individual mosquitoes from Ruili and Gengma counties, following which we performed assays to detect the S989P, V1016G and F1534C mutations in the *VGSC* gene (Additional file 2: Figure S1). Overall, V1016G showed the highest individual mutation rate (100.0%, 451/451), followed by S989P (81.6%, 368/451) and F1534C (78.9%, 356/451). Four mutation types were also observed in the tested samples: V1016G, V1016G + S989P, V1016G + F1534C and V1016G + S989P + F1534C. In the 209 *Ae. aegypti* samples from Ruili County, three mutation types were detected; the combinations and frequencies of these mutations were: V1016G + S989P (24.4%, 51/209), V1016G + F1534C (11.0%, 23/209) and V1016G + S898P + F1534C (64.6%, 135/209) (Fig. 3a). In contrast, four types of mutations were identified in the 242 samples from Gengma County: V1016G (0.4%, 1/242), V1016G + S989P (12.8%, 31/242), V1016G + F1534C (29.3% (71/242) and V1016G + S898P + F1534C (57.4%, 139/242) (Fig. 3b). The constituent ratio of *VGSC* gene mutation types in *Ae. aegypti* mosquitoes from Ruili and Gengma was significantly different (Fisher’s exact test, *P* = 0.000).

Four* kdr* mutation types were observed in *Ae. aegypti* mosquitoes from Ruili and Gengma counties, resulting in the identification of 12 genotypes. Among these genotypes, the combination S989P + V1016G + F1534C was particularly prevalent, accounting for 60.8% of the observed genotypes. The dominant mutation type was SP + GG + FC (31.4%). Additionally, two genotypes of S989P + V1016G were identified, accounting for 20.8% of the observed genotypes, with PP + GG being the primary type (97.9%). Also, three genotypes of V1016G + F1534C were detected, accounting for 18.19% of observed genotypes, with GG + CC being the primary type (85.4%). Only one single mutation genotype of V1016G was identified as GG. The triple mutation of S989P + V1016G + F1534C was found to be dominant in both Ruili and Gengma counties, accounting for 64.6% and 57.4%, respectively. Althoug S989P + V1016G and V1016G + F1534C combined mutations were found at both locations, the V1016G + F1534C combination was higher in Ruili County, whereas the S989P + V1016G combination was higher in Gengma County (Fig. 3a, b). These findings indicate that the mutation types of *Ae. aegypti* varied at the different locations, and that a comprehensive understanding of these mutations could be helpful in devising effective strategies for controlling vector-borne diseases.

### Association between *kdr* gene mutations and resistant phenotypes

To determine the impact of *kdr* mutations on resistance to the three pyrethroid insecticides, we first analyzed them separately for their associations with 3.20% permethrin, 0.08% lambda-cyhalothrin and 0.20% deltamethrin. Regarding exposure to 3.20% permethrin, we found that the frequency of the F1534C mutation in mosquitoes from Ruili County was significantly higher in resistant mosquitoes than in susceptible ones (Fisherʼs exact test,* P* = 0.01, OR = 7.43, 95% CI 1.71–32.29). Regarding exposure to 0.08% lambda-cyhalothrin, we found that the frequency of the F1534C mutation in mosquitoes from Gengma County was significantly higher in resistant mosquitoes than in susceptible ones (Chi-square test, *χ*^*2*^ = 9.29, *df* = 1, *P* < 0.001). Overall, the F1534C mutation was found to provide increased protection against insecticides, whereas the S989P and V1016G mutations were found not to have a significant relationship with the insecticies (Table 2). Generalized linear model analysis was used to reveal the relationship between the frequencies of mutation types and resistance to each insecticide. The results showed that triple mutations conferred *Ae. aegypti* from Gengma County with significantly greater resistance (Chi-square test, *χ*^2^ = 2.86, *df* = 1, *P* = 0.02) when exposed to 0.08% lambda-cyhalothrin; however, the triple mutation did not show a significant association with 3.20% permethrin and 0.20% deltamethrin resistance (*P* > 0.05) (Table 3).

**Table 2:** Correlation between the single *kdr* mutations S989P, V1016G and F1534C in *Ae. aegypti* from Ruili and Gengma counties with their resistance phenotypes to the three insecticides

Insecticide	Sampling site	Phenotype^a^	Number of mosquitoes tested	Single mutations							
				S989P			V1016G		F1534C		
				Frequency, *N* (%)	OR (95% CI)	*P* value (Fisher’s exact test)	Frequency, *N* (%)	Statistical analysis	Frequency, *N* (%)	OR (95% CI)	*P* value (Fisher’s exact test)
Permethrin (3.2%)	Ruili	R (Alive)	57	42 (73.7)	0.56 (0.11–2.86)	0.74	57 (100.0)	–^b^	52 (91.23)	7.43 (1.71–32.29)	0.01*
		S (Dead)	12	10 (83.3)			12 (100.0)		7 (58.33)		
	Gengma	R (Alive)	33	28 (84.9)	_	0.57	33 (100.0)		24 (72.73)	5.33 (0.83–34.34)	0.16
		S (Dead)	6	6 (100.0)			6 (100.0)		2 (33.33)		
Lambda-cyhalothrin (0.08%)	Ruili	R (Alive)	61	45 (73.8)	1.69 (0.36–7.88)	0.80	61 (100.0)		55 (90.16)	_	1.00
		S (Dead)	8	5 (62.5)			8 (100.0)		8 (100.00)		
	Gengma	R (Alive)	60	48 (80.0)	0.12 (0.0.02–0.98)	0.046*	60 (100.0)		52 (86.67)	9.29 (3.38–25.50)	0.00*
		S (Dead)	34	33 (97.1)			34 (100.0)		14 (41.18)		
Deltamethrin (0.2%)	Ruili	R (Alive)	53	39 (73.6)	0.16 (0.02–1.35)	0.12	53 (100.0)		50 (94.34)	4.76 (0.95–23.82)	0.12
		S (Dead)	18	17 (94.4)			18 (100.0)		14 (77.78)		
	Gengma	R (Alive)	0	0	_	_	0 (100.0)		0 (0.00)	_	_
		S (Dead)	109	95 (87.2)			109 (100.0)		78 (71.56)		
Total	Ruili	R (Alive)	171	126 (73.7)	0.53 (0.21–1.34)	0.17	171 (100.0)		157 (91.81)	3.48 (1.38–8.79)	0.01*
		S (Dead)	38	32 (84.2)			38 (100.0)		29 (76.32)		
	Gengma	R (Alive)	93	76 (81.7)	0.50 (0.24–1.06)	0.07	93 (100.0)		76 (81.72)	2.62 (1.40–4.87)	0.00*
		S (Dead)	149	134 (89.9)			149 (100.0)		94 (63.09)		

*CI* Confidence interval,* OR* odds ratio

*Statistically significant difference at *P* < 0.05

^a^S indicates susceptible, based on a mortality rate of ≥ 98%; R indicates resistance, based on a mortality rate  < 90%

^b^–, Not determined

**Table 3:** Correlation between the *kdr* mutation types in *Ae. aegypti* from Ruili and Gengma counties with their resistance phenotypes to the three insecticides

Insecticide	Sampling site	Phenotype^a^	Number of mosquitoes tested	Mutation type										
				S989P + V1016G + F1534C			S989P + V1016G			V1016G + F1534C			V1016G	
				Frequency, *N* (%)	OR (95% CI)	*P* value (Fisher’s exact test)	Frequency, *N* (%)	OR (95% CI)	*P* value (Fisher’s exact test)	Frequency, *N* (%)	OR (95% CI)	*P* value (Fisher’s exact test)	Frequency, *N* (%)	Statistical analysis
Permethrin (3.20%)	Ruili	R (Alive)	57	37 (64.9)	2.59 (0.73–9.22)	0.24	5 (8.8)	0.14 (0.03–0.59)	0.01*	15 (26.2)	1.79 (0.35–9.10)	0.74	0	_^b^
		S (Dead)	12	5 (41.7)			5 (41.7)			2 (16.7)			0	
	Gengma	R (Alive)	33	19 (57.6)	2.71 (0.43–16.96)	0.52	9 (27.3)	0.19 (0.03–1.21)	0.16	5 (15.2)	_	0.57	0	
		S (Dead)	6	2 (33.3)			4 (66.7)			0			0	
Lambda cyhalothrin (0.08%)	Ruili	R (Alive)	60	39 (65.0)	1.48 (0.36–6.13)	0.86	6 (10.0)	_	1.00	15 (25.0)	0.42 (0.10–1.76)	0.41	0	
		S (Dead)	9	5 (55.6)			0			4 (44.4)			0	
	Gengma	R (Alive)	60	40 (66.7)	2.86 (1.20–6.81)	0.02*	8 (13.3)	0.12 (0.04–0.33)	0.00*	12 (20.2)	_	0.01*	0	
		S (Dead)	34	14 (42.4)			19 (57.6)			0			1(2.94)	
Deltamethrin (0.20%)	Ruili	R (Alive)	53	36 (67.9)	0.81 (0.25–2.66)	0.73	3 (5.7)	0.21 (0.04–1.05)	0.11	14 (26.4)	6.10 (0.74–50.20)	0.12	0	
		S (Dead)	18	13 (72.8)			4 (22.2)			1 (5.6)			0	
	Gengma	R (Alive)	0	0	_^b^	_	0	_	_	0	_	_	0	
		S (Dead)	109	64 (58.7)			31 (28.4)			14 (12.8)			0	
Total	Ruili	R (Alive)	170	112 (65.8)	1.34 (0.66–2.74)	0.42	14 (8.2)	0.30 (0.12–0.75)	0.02*	44 (25.9)	1.59 (0.66–3.88)	0.30	0	
		S (Dead)	39	23 (59.0)			9 (23.1)			7 (18.0)			0	
	Gengma	R (Alive)	93	59 (63.4)	1.50 (0.88–2.55)	0.14	17 (18.3)	0.39 (0.21–0.73)	0.00*	17 (18.3)	2.16 (1.01–4.62)	0.04*	0	
		S (Dead)	149	80 (54.1)			54 (36.5)			14(9.5)			1(0.67)	

*CI* Confidence interval,* OR* odds ratio

*Statistically significant difference at *P* < 0.05

^a^S indicates susceptible, based on mortality rate of ≥ 98%; R indicates resistance, based on a mortality rate  < 90%

^b^–, Not determined

## Discussion

*Aedes aegypti,* commonly known as the yellow fever mosquito, has been identified as a significant transmission vector of DF since its first recorded presence in Ruili County in Yunnan Province in 2002 [30]. Over the years, the species has caused numerous local DF outbreaks in border areas such as Ruili and Dehong prefectures [31]. Consequently, the use of insecticides for controlling the *Ae. aegypti* vector is crucial for preventing DF in the region. However, the overuse and misuse of insecticides have led to the development of resistance in these mosquito populations, rendering these control measures increasingly ineffective [32, 33]. To tackle this issue effectively, it is crucial to have a clear understanding of the resistance status of *Ae. aegypti* populations in the region to the various insecticides being used. This study aimed to investigate the resistance of *Ae. aegypti* to the three main insecticides used in the two border counties of Ruili and Gengma in Yunnan Province. The results showed that the *Ae. aegypti* populations from both Ruili and Gengma exhibited widespread resistance to all three insecticides, with the exception of *Ae. aegypti* from Gengma County, which showed sensitivity to 0.20% deltamethrin. The development of resistance in mosquitoes can be attributed to the extensive use of these insecticides due to outbreaks of imported and local DF and the rapid increase in population density in both regions. To address this challenge, new strategies for vector control are urgently needed, including the use of alternative insecticides and implementation of novel approaches.

The resistance of *Ae. aegypti* to different insecticides is an important indicator for evaluating the effectiveness of insecticides. In our study, we found that the resistance of *Ae. aegypti* from two different locations, Ruili and Gengma counties, to 3.20% permethrin was similar, with a mortality rate of 18.1% and 21.9%, respectively. We also found that the mortality rate of *Ae. aegypti* from Ruili County exposed to 0.08% lambda-cyhalothrin was significantly lower than that of *Ae. aegypti* from Gengma County, indicating that *Ae. aegypti* from Ruili County has a higher level of resistance to this insecticide than those from Gengma County. This was also observed for 0.20% deltamethrin. Previous studies have also shown that *Ae. aegypti* from Ruili County have developed resistance to 0.03% lambda-cyhalothrin, with a mortality rate of 20.1% [32]. These results suggest that *Ae. aegypti* in different locations have developed varying levels of resistance to different insecticides. It should be noted that the concentration of insecticides used in various earlier studies is inconsistent, which limits the horizontal comparison of resistance levels and can only provide a reference level for resistance. Furthermore, there is a deficiency in the systematic monitoring of *Ae. aegypti* resistance at established monitoring points, which hinders the provision of comprehensive information on the evolution of resistance levels over time. Therefore, we recommend that local public health authorities systematically monitor insecticide resistance in different *Ae. aegypti* populations to provide a basis for practical decision-making regarding control measures for vector-borne diseases.

Several *kdr* mutations have been identified in *Ae. aegypti*, including V410L, G923V, L982W, S989P, A1007G, I1011M/V, V1016G/I, T1520I, F1534C/L and D1763Y [16, 34]. Previous research has demonstrated that specific individual mutation sites, such as V1016G and F1534C, can directly confer resistance to pyrethroids [35] and that combinations of double and triple mutations can further augment the level of resistance [16, 35]. Our analysis of 451 individual mosquitoes revealed mutation rates of 81.6% for S989P, 78.9% for F1534C and 100.0% for V1016G. In 2015, Li et al. [19] reported an 81.9% occurrence of the S989P mutation in *Ae. aegypti* in southern China, and in 2017, Shi Q-M et al. [36] found a 25.0% occurrence of the F1534C mutation in mosquitoes in Yunnan Province, China. These findings indicate that the S989P and F1534C mutations are prevalent mutations in *Ae. aegypti* populations in Yunnan and other regions of China. Notably, in 2017, Shi C-N et al. [28] observed the V1016G mutation in all 100 samples from a population in Yunnan Province, and in 2020, Lan et al. [37] reported a 100.0% mutation rate in four counties in Yunnan Province, with a mutation rate of 99.3% in a fifth county. These results suggest that the V1016G mutation has become a fixed trend in these populations. In addition, the distribution and origin of *kdr* mutations demonstrate unique global geographical characteristics. For example, the *kdr* mutation combination S989P + V1016G was found exclusively in Asia, while the F1534C mutation was present in samples from almost all continents [38, 39]. Understanding the distribution and origin of *kdr* mutations is crucial for developing effective strategies to combat insecticide resistance and control vector-borne diseases [40].

Our study found four mutation types and 12 genotypes in the tested *Ae. aegypti* specimens, with triple, double and single mutations accounting for 60.8%, 36.8% and 0.2% of all mutations, respectively. The most common genotypes were SP + GG + FC and PP + GG. According to Moyes et al. [41], the presence of two mutations (S989P + V1016G/V1016G + F1534C) could enhance the resistance of *Ae. aegypti* to pyrethroids. The presence of a triple mutation (S989P + V1016G + F1534C) has been linked to an even higher insecticide resistance, with the authors of one study reporting a resistance level that was 28-fold [42] and 90-fold that of the sensitive strain [24]. Additionally, high frequencies of 1520I + 1534C and T1520 + 1534C in *Ae. aegypti* from Pakistan have been found to confer resistance to pyrethroids [43]. These results suggest that multiple mutations have a strong synergistic effect and that combined mutations are more effective than a single mutation in conferring resistance to pyrethroids. Monitoring changes in the category and intensity of resistance is crucial for responding to DF epidemics and for their prevention and control, as well as for providing important references and evidence for the control of other vector-borne diseases.

Although this study provides valuable insight into the resistance of *Ae. aegypti*, certain limitations must be taken into consideration. Firstly, the *VGSC* gene comprises multiple loci, and only the three most common ones were studied in this research. The contribution of the remaining loci to resistance needs to be explored further. Additionally, other mechanisms related to resistance, such as enzymatic metabolism, have not yet been systematically studied. Secondly, the study only measured resistance to those insecticides commonly used for dengue vectors. In contrast, the potential effects of interactions and resistance levels of other insecticide classes, particularly agricultural insecticides, were not considered. Moreover, due to the rapid expansion of this invasive mosquito species, it is essential to conduct vector resistance surveillance (including bioassay and genetic assay) in other nearby regions, including both endemic and non-endemic countries, to provide comprehensive and systematic evaluation indicators for the prevention and control of DF in Yunnan Province.

## Conclusions

In conclusion, this study provides substantial preliminary data on the resistance of *Ae. aegypti* to insecticides commonly used for dengue vector control. The co-occurrence of the *kdr* mutations resulted in a higher resistance level and conferred a complex resistance pattern. The development of resistance to synthetic pyrethroid insecticides in the *Ae. aegypti* populations from the two endemic areas studied here highlight the need for continuous monitoring of insecticide resistance and the rational selection of insecticides. Further research into the mechanism of insecticide resistance in vector mosquitoes is essential for effective decision-making and the development of novel strategies for vector control.

### Supplementary Information

**Additional file 1: Table S1.** Allele-specific PCR (AS-PCR) primer for *Ae. aegypti.*

**Additional file 2: Fig. S1.** AS-PCR results of knockout resistance gene mutation in *Ae. aegypti*. **a** heterozygote and homozygote of S989P mutants, **b** heterozygote and homozygote of V1016G mutants. **c** Heterozygote and homozygote of F1534C mutants; M, DNA marker; SS, susceptible homozygote; RS, resistant heterozygote; RR, resistant homozygote.

## Data Availability

All data generated or analyzed during this study are included in this published article and its supplementary information files.

## Abbreviations

AS-PCR: Allele-specific PCR
DF: Dengue fever
*kdr*: Knockdown resistance gene
KDT_50_: Time taken for 50% of the test mosquitoes to be knocked down
VGSC: Voltage-gated sodium channel gene
CI: Confidence interval
OR: Odds ratio
*χ*^2^: Pearson’s Chi-square test
