# Supplementary material for: Pyrethroid resistance status and co-occurrence of V1016G, F1534C and S989P mutations in the Aedes aegypti population from two dengue outbreak counties along the China-Myanmar border
Source: Parasit Vectors. 2024 Feb 27;17:91. doi: 10.1186/s13071-024-06124-9 (PMC10898090; doi:10.1186/s13071-024-06124-9)
Supplement: Supplementary file 1 — Additional file 1: Table S1. Allele-specific PCR (AS-PCR) primer for Ae. aegypti. [file 13071_2024_6124_MOESM1_ESM.docx]

Additional file 1: Table S1 Allele-specific PCR (AS-PCR) primer for *Aedes aegypti*

| Mutation site | Primer | Primer sequences. （5’-3’） | Product length（bp） |
| --- | --- | --- | --- |
| S989P | M1-For | AGACAATGTGGATCGCTTCC | 635 |
|  | M1-Rev | GGACGCAATCTGGCTTGTTA |  |
|  | M1-S | GCATACAATCCCACATGGA | 129 |
|  | M1-P | GCATACAATCCCACATGGG | 129 |
| V1016G | M2-For | GCCACCGTAGTGATAGGAAATC | 592 |
|  | M2-Rev | CGGGTTAAGTTTCGTTTAGTAGC |  |
|  | M2-V | GTTTCCCACTCGCACAGGT | 348 |
|  | M2-G | GTTTCCCACTCGCACAGGG | 348 |
| F1534C | M3-For | GGAGAACTACACGTGGGAGAAC | 517 |
|  | M3-Rev | CGCCACTGAAATTGAGAATAGC |  |
|  | M3-F | GCGTGAAGAACGACCCGA | 284 |
|  | M3-C | GCGTGAAGAACGACCCGC | 284 |
