# Supplementary figures and images for: Pyrethroid resistance status and co-occurrence of V1016G, F1534C and S989P mutations in the Aedes aegypti population from two dengue outbreak counties along the China-Myanmar border
Source: Parasit Vectors. 2024 Feb 27;17:91. doi: 10.1186/s13071-024-06124-9 (PMC10898090; doi:10.1186/s13071-024-06124-9)

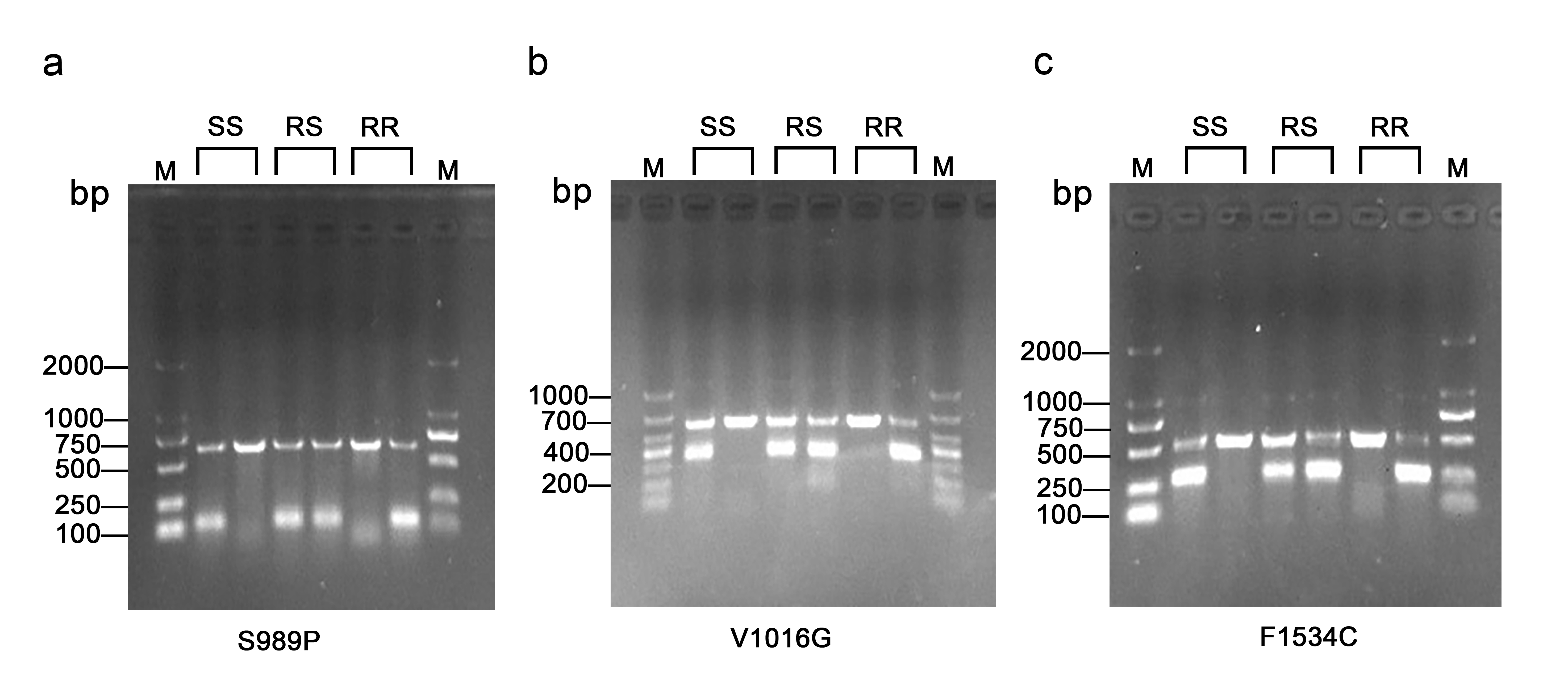

Supplement: Supplementary file 2 — Additional file 2: Fig. S1. AS-PCR results of knockout resistance gene mutation in Ae. aegypti. a heterozygote and homozygote of S989P mutants, b heterozygote and homozygote of V1016G mutants. c Heterozygote and homozygote of F1534C mutants; M, DNA marker; SS, susceptible homozygote; RS, resistant heterozygote; RR, resistant homozygote. [file 13071_2024_6124_MOESM2_ESM.tif]
